# Young Children and Voice Search: What We Know From Human-Computer Interaction Research

**DOI:** 10.3389/fpsyg.2019.00008

**Published:** 2019-01-22

**Authors:** Silvia B. Lovato, Anne Marie Piper

**Affiliations:** Communication Studies, Northwestern University, Evanston, IL, United States

**Keywords:** children, question-asking, voice interfaces, internet search, information-seeking

## Abstract

Young children are prolific question-askers. The growing ubiquity of voice interfaces (e.g., Apple’s Siri, Amazon’s Alexa), as well as the availability of voice input in search fields, now make it possible for children to ask questions via Internet search when they are able to speak clearly, but before they have learned to read and write, typically between 3 and 6 years of age. The prevalence of *voice search* makes it important to understand children’s changing conceptions of digital devices as a source of information and the role of technology-mediated question-asking in development. While limited research has focused on young children’s use of voice interfaces, reviewing two related bodies of literature sheds light on how this use might unfold. This paper brings together studies of how children look for information, and of how they perceive and understand the informational and social roles of technology, drawing on human-computer interaction research. We conclude by highlighting lines of questioning for future work on younger children’s interaction through voice search.

## Voice Search and Children’s Questions

Young children are curious and prolific question-askers. They are known to ask factual and causal questions about the world around them when they perceive a gap in their understanding (Tizard and Hughes, 1984; Callanan and Oakes, 1992; Chouinard et al., 2007). Voice interfaces powered by natural language processing, such as Apple’s Siri, Amazon’s Alexa, and the Google Assistant, as well as the availability of microphone input features on the search fields of Google, YouTube, and other services make it possible for children to press a button, or use a “wake word,” and simply ask a question or perform an Internet search. The present paper refers to this interaction as *voice search*.

Voice search is now a common part of many interfaces on traditional computers, connected home speakers, and mobile devices. Smartphones and tablet computers in particular have become ubiquitous in the lives of American children. According to a report from Common Sense Media (Rideout, 2017), almost all (98%) American children now live in a home with a tablet or smartphone, and this trend includes low-income families adopting technology at similar rates (Kabali et al., 2015). Unlike keyboards and mice, touch screens are immediately intuitive and operated with gestures such as pointing and swiping, which develop in the first year of life. Indeed, children aged 12 to 17 months are able to navigate simple tablet-based applications with moderate ability (Hourcade et al., 2015).

While text-based Internet search results might not be accessible to pre- and emerging readers, the explosive growth of online video content supports young children’s ability to independently find and consume online information. Robert Kyncl, YouTube’s Chief Business Officer, predicted at the 2012 Consumer Electronics Show that by 2020, 90% of Internet traffic would be used by video, a prediction later anticipated by Cisco to 2019 (Tribbey, 2016). Google search results come with a video tab, from which children can choose a video based on a representative picture, press the play button and watch a video related to their query. Thus, this dramatic shift in young children’s ability to search online is due to both the prevalence of voice-based, natural language search features and the increasing volume of video-based search results.

This shift in children’s ability to find information through connected devices makes it important to understand their changing conceptions of digital devices as sources of information and how they might fare as they attempt to use them to find answers to their questions. However, research has yet to understand the behaviors of young children using voice search. By young children, here, we refer to children who are able to speak fully formed sentences but have yet to learn how to read and write with enough fluency to perform internet searches by typing, typically between the ages of 3 and 6 years.

To help bridge this gap and identify promising lines of future work, this review examines the existing literature on children’s search behavior as well as studies of children’s perceptions of technology. These existing studies largely focus on children ages 7 and older, because until recently, searching had required reading, writing and typing skills, making it out of reach for younger children. However, the findings of these studies can help shed some light on what might happen when younger children attempt to ask questions of technology independently.

We start by reviewing studies that focus on how children as young as age 7 have searched the Internet at various points during its history, including child-specific web directories like Yahooligans (Bilal, 2000, 2001, 2002), keyword searches (Druin et al., 2009, 2010) and the more recent use of natural language (Kammerer and Bohnacker, 2012). To complement this literature, we review studies of how children understand technology, including their ideas about computers in general (e.g., Van Duuren et al., 1998; Rücker and Pinkwart, 2016 for a comprehensive review), how they understand robots (Kahn et al., 2012, 2013) and media technology (Reeves and Nass, 1996; Chiasson and Gutwin, 2005). We include these studies because children’s perceptions of technology impact their expectations of whether such technology might serve as sources of information. We end by putting what we know in the context of child development and suggesting new areas for future research and design involving developmentally appropriate interactions through voice search.

## Children and Internet Search

Studies of how children aged 7 and older search for information in digital interfaces began with the CD-ROM encyclopedias and digital libraries of the 1980s and 1990s, where the realm of information available was limited (e.g., Marchionini, 1989). Even then, elementary-aged children showed a tendency to use natural language in search fields (Marchionini, 1989). In a system that was designed to find keywords, this strategy failed, generating no results (Solomon, 1993).

In a series of studies of seventh graders using the web directory Yahooligans, a child-focused resource managed by Yahoo, Inc., from 1996 to 2006, Bilal (2000, 2001, 2002) found that children consistently preferred to browse the directory than to use the search functionality. Only 50% of the students succeeded at finding answers to specific, fact-based queries given by a science teacher, while 69% partially succeeded at researching a topic more generally using their own queries and 73% succeeded at finding answers to an undirected, self-generated query. Bilal (2002) also reported that 13% of children in the third study, who were using their own queries, used natural language instead of keywords, something seen as a liability at the time, leading to the conclusion that students should receive better web search training.

In a more recent study about how children ages 7, 9, and 11 used keyword interfaces to search the Internet (Druin et al., 2009), the researchers found that children had trouble typing, spelling and deciding which words to use as search terms. Specifically, children tended to look at the keyboard while typing, making it difficult to catch typos until the entire word or phrase had been entered and to see the predictive terms offered by the search engine. Parents in their study suggested voice-input as a solution to children’s typing and spelling problems. The study also found other difficulties that might not be eliminated by voice input: for example, children had difficulty choosing which words to use and breaking down a complex query into multiple steps when needed (query reformulation). When asked to find what day of the week the vice-president’s birthday would fall on the following year, none of the children were able to find the answer; the youngest children, age 7, did not even try.

In a larger study including 83 children, again aged 7, 9, and 11, and their parents (Druin et al., 2010), these findings were confirmed and expanded: the researchers identified seven distinct search “roles,” or search behavior patterns, displayed by the children, in isolation or combined with one or more other roles. Each of these roles is associated with specific behaviors, triggers (motivation for using search), obstacles (such as typing, spelling and reading difficulties, lack of motivation and self-imposed limiting rules) and influencer, or parent, roles (demonstrator, fixer, mentor). The most common role was that of a *developing* searcher, displayed by 58 of the children. Developing searchers were found to be willing to search but possess a limited command of search tools and, again, a tendency to use natural language. The developing role was most often displayed at the same time as that of *domain-specific* searcher, in which children are comfortable with a few “tried-and-true” resources, usually related to personal interests, and tend to return to those websites repeatedly, even when searching for unrelated information. For example, children attempted to find information about dolphins and about the vice-president of the United States at a games website and on spongebob.com. Other roles identified were *power searcher, distracted, non-motivated, visual*, and *rule-based searcher*.

While Druin et al. found that children’s use of natural language in search engines was problematic, Kammerer and Bohnacker (2012) compared natural language to keyword searches performed by 21 children aged 8 to 10 using Google in German and found that natural language users were more successful than those using keywords. They gave children a two-part task in which the first part was a simple yes/no question (do all kangaroos have pouches?) and the second required a more complex strategy and answer (how do baby kangaroos stay in pouches?). Tasks were given orally and children could choose what to enter in the search field. Of the 13 natural language users, 8 were able to answer both parts of the task correctly, 4 were able to answer only the first and one was unable to answer either. The 8 keyword users fared far worse, with only 3 being able to answer both queries correctly, 3 answering only the first and 2 being unable to answer either.

As we consider younger children using voice interfaces to search, some of the mechanical obstacles identified by prior work (e.g., typing and spelling difficulties) may lose importance while the discrepancy between the intended users of the interface, by and large adults, and younger children, who now have access to search, increases. For example, Druin’s *domain-specific* searchers might become *app-specific* in this generation. Young children who become comfortable searching inside an application such as *YouTube Kids* could attempt to use it for queries that would be better served by a different tool. Younger users also have a less developed vocabulary and may be less precise in how they formulate queries. Additionally, while videos and spoken responses may dispense with reading requirements, such audio and video content was likely not produced with young children in mind, creating the potential for comprehension difficulties. These obstacles, however, only matter if younger children indeed perceive these technologies as sources of information and attempt to ask questions of them.

## Children’s Perceptions of Digital Devices

To predict whether young children might see the devices they use as potential sources of answers to their questions and not just game and video players, we consider how they conceptualize computing devices. Existing work (Van Duuren et al., 1998; Papastergiou, 2005; Yan, 2005; Diethelm et al., 2012; see Rücker and Pinkwart, 2016 for a review) about older children’s understanding of computers and the Internet has found that they perceive computers as capable of containing infinite amounts of information (e.g., Van Duuren et al., 1998); however, results of a few recent studies (McKenney and Voogt, 2010; Eisen and Lillard, 2016, 2017) with younger children have been mixed.

Rücker and Pinkwart (2016) present a systematic, interdisciplinary review of studies of children’s conceptions of computers. They identify five main ideas children have expressed in studies over the years, between 1968 and 2012: (1) intelligent machines; (2) omniscient databases; (3) mechanical devices; (4) wire networks and (5) programmable machines. As we consider the notion of computer-like devices as information sources to young children, the most relevant concepts are those of an intelligent machine and an omniscient database. Studies included in the review found that children aged 8 and 11 (but not 5-year-olds) believed computers had the results of all possible mathematical calculations already stored in their memory (Van Duuren et al., 1998) and that 12-to-16 year-olds believed that the entire Internet was stored in one single computer, either the user’s own or another accessible through the network (Papastergiou, 2005; Diethelm et al., 2012).

Studies with younger children, however, present a more mixed picture. A study of Dutch children’s perceptions of their own computer use including 4- to 7-year-olds, most of whom had daily access to computers both in and out of school, found that the overwhelming majority of young children used computers to play games and that using the computer for a creative or communicative activity or to search the Internet was far less common (McKenney and Voogt, 2010). Eisen and Lillard (2016, 2017) performed two studies to understand which functions preschool children attribute to touch screen devices when compared to other media such as television and books. In the first study, they found that children tend to attribute fewer functions to most objects than adults. When asked to identify the best object for learning about dogs, hearing Spanish or looking at a map, touch screen devices were not their top choice. The computer was the preferred method for seeing a map. In the second study, children were asked to choose between a tablet computer and a book for several learning tasks (e.g., cooking, the weather, Virginia, yesterday’s football game). While the younger children in the sample showed no clear preference, 6-year-olds preferred the tablet computer for most tasks. However, children did not take into account whether the information sought was timely (i.e., the weather, yesterday’s football game), with even 6-year-olds preferring books to learn about the game.

While voice input is available, for example via the Google mobile application, as well as YouTube and YouTube Kids, these interfaces don’t respond verbally, but show the user’s query as text input in the search field and then display search results after the query is submitted. Conversational agents (like Siri or Alexa), on the other hand, are programmed to respond as a person would. Research on how children understand and interact with robots and with other media provides insights into how machines that attempt to act like humans are perceived by children.

Kahn et al. (2013) argue that social robots are establishing a new ontological category, distinct from humans, animals or simple artifacts. As children interact with a social robot, they tend to believe that it has rights and feelings (Kahn et al., 2012). At the same time, they are aware of the robot’s machine status. Through a number of experiments, Reeves and Nass (1996) found that people tend to respond to computers and other media as they would to humans. They refer to this phenomenon as *the media equation.* The set of interactions that are specific to computers, whose responses, unlike those of television, are contingent on user input, are studied under an area of research called CASA (Computers as Social Actors). But is the tendency the same in children? Some critics of these theories argue that only inexperienced users would respond to machines as if they were people. Children, then, could easily be expected to act this way. Chiasson and Gutwin (2005) predicted that children would be even more affected by the media equation than adults, since they are more likely to anthropomorphize objects and accept fictional characters as real. They also predicted that providing social cues in interfaces that made interactions closer to those with people would help children stay engaged in educational activities. To test this, they replicated two classic Nass and Reeves CASA experiments comparing groups of adults to children aged 10 to 12. In both experiments, they measured the impact of social language – praise in one case and treating the participant as part of the computer’s team in another – on users’ assessments of their own experiences playing simple games. Surprisingly, they found that, while social language had a positive impact on adults, it had no impact on the children. They proposed two explanations for this: one is that children are so affected by the media equation that this overwhelms any difference between experimental conditions (i.e., they would have had a positive experience regardless of the social language in the game). The other explanation is that people who have grown up with computers, as was the case of the child participants, are less susceptible to the media equation than those who learned to use computers later in life, as was the case of the adult participants.

## Voice Search in the Context of Development: Directions for Future Research

While voice input removes mechanical obstacles to Internet searching, such as typing and spelling, there are other developmental factors to take into account as we consider younger children using voice search. First, children who are able to make themselves understood by language processing software are still developing theory of mind skills, broadly defined as the set of skills that allows us to understand the mental states of others. From our own prior work (Lovato and Piper, 2015), we know that one of the obstacles young children face when using voice search is not fully understanding what the system can and cannot answer (i.e., what the system knows) and how much context to provide. For example, systems cannot usually answer questions about the location of specific people or objects – at least not yet – and cannot answer questions about undescribed objects or referents it cannot see (e.g., “where was this made?”). Understanding what someone knows is an aspect of theory of mind that is still in development in young children (Wellman and Liu, 2004).

Preschoolers’ trust in technology sources has been found to be largely based on previous experience, as it is with people (Danovitch and Alzahabi, 2013). This behavior evolves with age, with 4- and 5-year-olds being more likely to use past experience as a reference than 3-year-olds (Mills et al., 2011). The imperfect ability of voice agents to understand children’s speech, combined with the agents’ inability to ask for more information or context, could have an impact on how much children learn to rely on conversational agents as sources: if Siri or Alexa misunderstands a child and responds with an answer that doesn’t make sense, the child might lose trust in it as a source of answers.

While the existing literature on older children’s Internet search and perceptions of technology as information sources seems to support the potential for younger children to use voice search, it also points to two central lines of inquiry regarding what happens when younger children ask questions of voice interfaces or conversational agents. The first relates to the distinct obstacles young children might face when using this technology and how voice interfaces can better support children in their developmental needs. The second, equally important question, relates to how the particular use of language required by search engines and conversational agents might shape how children learn to use language to obtain information.

As mentioned, young children ask questions when they perceive an inconsistency, or a gap, in their understanding of the world (Tizard and Hughes, 1984; Callanan and Oakes, 1992; Chouinard et al., 2007). Chouinard et al. (2007) found that children’s levels of persistence in question-asking are high when they receive responses that do not contain the information requested and low when they do receive such information, suggesting that children really are looking for information (as opposed to simply adult attention). In understanding young children’s goals when asking information-seeking questions (i.e., filling gaps in understanding), it is important to consider what an optimal answer would be: would a piece of information stated in a way the child can understand suffice? Or is a conversation indispensable?

It is possible that when children ask questions, at least some of the time, a simple factual answer is not the best answer. When children direct factual questions at adults, these serve as “more knowledgeable others,” who help children advance their state of development (Vygotsky, 1978). Parents and teachers might ask a child why she is asking a question, or what she thinks the answer is, scaffolding the child as she figures out the answer, partly on her own. Through dialog, children not only develop understanding, but also language and reasoning. Can conversational agents serve as more knowledgeable others? Future research should consider how child-friendly conversational agents should respond to children’s queries for optimal child development outcomes.

There is no question that voice search and conversational agents will continue to develop. It is not impossible for this technology to be made more child-friendly by, for example, learning to distinguish between child and adult voices and responding to children in ways that are more supportive. A system could explain what it cannot answer or request additional information in order to respond to a query. Such developments could encourage young children to use these systems more frequently, in turn increasing our need to understand how such use could impact language development and cognitive development more broadly.

## Author Contributions

All authors shared the research, writing, and editing involved in producing this article.

## Conflict of Interest Statement

The authors declare that the research was conducted in the absence of any commercial or financial relationships that could be construed as a potential conflict of interest.
